# What to Expect When the Unexpected Becomes Expected: Harmonic Surprise and Preference Over Time in Popular Music

**DOI:** 10.3389/fnhum.2021.578644

**Published:** 2021-04-30

**Authors:** Scott A. Miles, David S. Rosen, Shaun Barry, David Grunberg, Norberto Grzywacz

**Affiliations:** ^1^Interdisciplinary Program in Neuroscience, Georgetown University, Washington, DC, United States; ^2^Secret Chord Laboratories, Norfolk, VA, United States; ^3^Music and Entertainment Technology Laboratory, Drexel University, Philadelphia, PA, United States; ^4^Department of Psychology, Loyola University Chicago, Chicago, IL, United States; ^5^Department of Molecular Pharmacology and Neuroscience, Loyola University Chicago, Chicago, IL, United States

**Keywords:** music, surprise, harmony, preference, predictive coding

## Abstract

Previous work demonstrates that music with more *surprising* chords tends to be perceived as more enjoyable than music with more conventional harmonic structures. In that work, harmonic surprise was computed based upon a static distribution of chords. This would assume that harmonic surprise is constant over time, and the effect of harmonic surprise on music preference is similarly static. In this study we assess that assumption and establish that the relationship between harmonic surprise (as measured according to a specific time period) and music preference is not constant as time goes on. Analyses of harmonic surprise and preference from 1958 to 1991 showed increased harmonic surprise over time, and that this increase was significantly more pronounced in preferred songs. Separate analyses showed similar increases over the years from 2000 to 2019. As such, these findings provide evidence that the human perception of tonality is influenced by exposure. Baseline harmonic expectations that were developed through listening to the music of “yesterday” are violated in the music of “today,” leading to preference. Then, once the music of “today” provides the baseline expectations for the music of “tomorrow,” more pronounced violations—and with them, higher harmonic surprise values—become associated with preference formation. We call this phenomenon the “Inflationary-Surprise Hypothesis.” Support for this hypothesis could impact the understanding of how the perception of tonality, and other statistical regularities, are developed in the human brain.

## 1. Introduction

In Miles et al. (2017), the examination of harmonic surprise and music preference tested two seemingly contradictory hypotheses about harmonic surprise and music preference. The *Absolute-Surprise Hypothesis* states that moderate increases in harmonic surprise are perceived as “good”: there is a relationship between music preference and musical popularity that is dependent on how much total harmonic surprise is contained in a piece of music. The *Contrastive-Surprise Hypothesis* states that increases in harmonic surprise are perceived as “bad”: sections of music with elevated harmonic surprise lead to a mild pain signal in the brains of listeners, and when this is relieved during a subsequent section with lower harmonic surprise, the result is pleasure, leading to music preference.

Evidence supporting both hypotheses led to the formulation of the Hybrid-Surprise Hypothesis. Analyses showed that the per-song average harmonic surprise of the top quartile (*Q*_1_) of Billboard charting songs is significantly higher than that of the bottom-quartile (*Q*_4_) songs, providing support for the *Absolute-Surprise Hypothesis*. Furthermore, the results revealed that there is increased variation in surprise across the sections of *Q*_1_ songs than *Q*_4_ songs, providing support for the *Contrastive-Surprise Hypothesis*. However, each of these analyses included songs across all 33 years of the corpus, without taking release date into account. The findings thus assumed uniform effects of surprise on preference, based on a distribution of chords that never varies. This uniformity is the simplest hypothesis.

It is possible, however, that there are significant changes over the years in either the effects of surprise, the underlying chord distributions, or both. In fact, the musical properties of popular music have been shown to evolve over time (Mauch et al., 2015). The songs examined in Miles et al. (2017) from the McGill Billboard corpus (Burgoyne et al., 2011) span 33 years in their release date (1958–1991). Thus, it is likely that the effects of absolute or contrastive surprise on preference measurably change over this span of years, and any such change must be accounted for to further understand the nature of music preference and harmonic surprise. Through a separate set of analyses, the present study is designed to address this gap in the understanding of the relationship between harmonic surprise and preference in popular music.

In addition to addressing this gap in understanding, we also set out to address a gap in time. The present study introduces the possibility of a dynamic relationship between harmonic surprise and preference as time goes by. This highlights the lack of recently released songs in the McGill Billboard Corpus, which ends in 1991. To see if any change in the effects of harmonic surprise on preference over time extend to the current era of popular music, we added an analysis of the Secret Chord Laboratories (SCL) corpus. This corpus features a nearly exhaustive list of Billboard-charting songs released from January 1, 2000 to December 31, 2019.

## 2. Literature Review

The process by which the human brain perceives music is being extensively studied by researchers in the field (McDermott et al., 2016; Reybrouck et al., 2018; Daly et al., 2020). One recent development of note is the determination that the content of music may matter less to a listener's perception of a musical work than whether or not the listener enjoys that work (Wilkins et al., 2015). In other words, a listener is likely to have a more similar cognitive reaction to hearing two pieces of music that he or she prefers, even if those two works are very different, than to hearing two similar pieces of music, one of which he likes and one of which he or she dislikes. As such, determining how the human brain determines whether a particular piece of music is enjoyable or not is of particular interest.

One prevailing theory as to how music evokes pleasurable responses in the human brain is that the music, by adhering to or deviating from what a listener would expect, can stimulate a neural reward (Meyer, 1956; Huron, 2006). This is reinforced by the evidence that music perception is based at least in large part on cultural knowledge. It has been shown that the musical culture that a listener grows up with has an influence on the understanding and perception of music later in life (Curtis and Bharucha, 2008), to the point where an individual's perception of, and enjoyment of, a new musical piece is heavily dependent on music already heard (McDermott et al., 2016). However, it has historically been difficult to evaluate this idea, in part because the concepts involved (such as the “amount of surprise” in the music or the expected “reward”) are difficult to quantify. A system that could precisely estimate the amount of “surprise” in a piece of music and how much people might be expected to like it would thus be of utility to the research community.

In order to estimate surprise, one approach is drawn from the field of information theory (Rohrmeier and Koelsch, 2012). This approach has proven useful at describing various aspects of music. However, until recently, information theory-based approaches have not been able to take the additional step of describing how those aspects influence the perception of a given musical work.

One potentially useful aspect of information theory is the concept of “surprise,” a measure of how much a given element deviates from what that element would be expected to be (Atick, 1992). This concept has been applied to music in order to determine how much a specific musical element deviates from the norm (Egermann et al., 2013). By taking a musical piece and calculating the amount of surprise in its components (such as its harmonies, its melody, its rhythm, its timbre, etc.), it could thus become possible to quantify the total amount of surprise in the music.

Furthermore, just as work has been done to quantify surprise, progress has also been made in quantifying musical perception. Because the position of a musical work on charts such as the Billboard Hot 100 is a function of how many people listen to it and buy it (Parker, 1992), music which delivers more reward to listeners can be expected to place higher on the charts than music which does not deliver as much reward. As such, features such as chart position can be used as an approximation of popularity and musical reward.

However, while there do exist projects which have sought to analyze musical response in terms of surprise, particularly the surprise of the music's harmonic content, much of this work relies on artificial datasets comprised of individual chords (Koelsch et al., 2001) or old, relatively simple music such as Bach chorales (Steinbeis et al., 2005), as opposed to modern songs. We therefore developed prior work on this topic (Miles et al., 2017) in which we calculated the harmonic surprise of actual popular music and assessed how this surprise related to the music's popularity on the charts. This was done to ensure that our results were relevant to the music people actually listen to in the modern age, or at least in the time since the Billboard Hot 100 began being published in 1958.

Our prior work on this subject considered two possible hypotheses as to how surprise affects musical perception. One is the *Absolute-Surprise Hypothesis*, which states that musical popularity is determined by the overall amount of surprise in a piece. This is based on the theoretical foundation that dopamine, a pleasurable brain chemical, is often associated with novelty (Suhara et al., 2001), and thus that listening to music which is surprising or novel will likely produce more of this chemical. In addition to our own work, another prominent paper in this field used functional magnetic resonance imaging (fMRI) and positron emission tomography (PET) machines to discover that harmonically unexpected elements of a work do tend to induce more dopamine production than more conventional musical elements (Salimpoor et al., 2011).

The second hypothesis we considered is the *Contrastive-Surprise Hypothesis*. In this approach, the response to a piece of music is not dependent on the total amount of surprise in the piece but on the contrast between high-surprise and low-surprise sections within a given musical work. This hypothesis is in line with previously-advanced theories noting that pleasure can be derived from first building up tension (such as with a surprising element) and then relieving it (as with a non-surprising element; Huron, 2006). Previous electrophysiological work (Koelsch et al., 2001) has found an association with the perception of unexpected chords to the neural correlate of mild irritation known as the early right anterior negativity, associated with prediction error being processed in the brain. The *Contrastive-Surprise Hypothesis* is consistent with a model of reward resulting from the relief of such irritation.

It is worth noting that our prior work (Miles et al., 2017) used a single distribution of chords over all time and thus assumed that the “expected” harmony of music was constant over the years across Western popular music. This assumption, however, may not have been valid; it is entirely possible that the common harmonies which can be reasonably expected to occur in music may change from year to year. As such, we present this current work, which seeks to investigate this possibility and determine if using a more sophisticated model for the expected harmonies allows for a more accurate model.

We also investigate the hypothesis that preferred music tends to increase in surprise over time, whether absolute, contrastive, or both, at a rate higher than any such increase over time in less preferred music. This hypothesis, which we call the *Inflationary-Surprise Hypothesis*, might be due to the ever-increasing requirement for music being released at any given time, considered “high-surprise,” to build upon the already increased surprise within preferred music that already exists. This phenomenon could be largely driven by effects of the listening habits during critical periods in the formation of harmonic expectations by listeners. The Billboard Hot 100 is known to be driven by an adolescent cohort of consumers. At this stage in their lives, teenagers generally want to be associated with the most popular new song or artist, since music preference is important to identity formation. In four studies, North and Hargreaves (1999) reported that music preference of a particular style functions as an “identity badge,” whereby adolescents form their self-concepts and social judgments. It appears that these personal music definitions and choices for adolescents are likely to elicit emotional or spiritual experiences (Bosacki and O'Neill, 2015). The heightened social and emotional impact of music for adolescents creates a strong nostalgia, rekindling images of past selves, experiences, and friends who shared those musical preferences. Furthermore, the emotional content and subject matter of popular music connect with adolescents, because its sound and lyrics match the extreme emotional experience of their daily lives (Wells and Hakanen, 1991). It has been reported that music can function therapeutically to reduce feelings of stress and loneliness in adolescents (Zillmann and Gan, 1997).

If the effect of absolute and contrastive surprise on music preference indeed increases over time, this may be due to cascading cohorts of primarily adolescent listeners whose baseline expectations have been formed during a critical period of statistical learning at an earlier age. Each of these successive cohorts could be driving an apparent effect whereby overall harmonic surprise of preferred songs, as measured against a constant distribution of chords from the past, increases over time. Evidence of the *Inflationary-Surprise Hypothesis* would also support broader theories about musical expectations being learned through exposure early in life. In his song about advancements as a result of human progress, “Boy in the Bubble,” Simon (1986) sings: “.every generation throws a hero up the pop charts.” It is possible that these “heroes” use increasing harmonic surprise, over time, in their songs.

## 3. Materials and Methods

The songs included in the McGill Billboard corpus of songs from 1958 to 1991 were separated into four consecutive time bins to examine how the effects of harmonic surprise on music preference change over time. The songs of a more recent corpus, a set of 6,051 songs on the Billboard Hot 100 chart released from 2000 to 2019 (the *SCL Corpus*), were also separated into four consecutive time bins (see Table 1). The *SCL Corpus* features a considerable representation of the 7,988 total unique songs that charted on the Billboard Hot 100 over that 20-year span of time. The null hypotheses stipulated that for each corpus, each type of harmonic surprise effect on preference across the four time bins would not significantly differ from one another. Support for this null hypothesis would suggest that there is no impact of time on the effects of harmonic surprise on music preference.

**Table 1:** Four time bins and corresponding periods within which songs of each time bin were released for both the “past” and “present” aspects of this study.

**Time bin**	**Period**
McGill 1	August 1958–January 1975
McGill 2	February 1975–July 1980
McGill 3	August 1980–January 1986
McGill 4	February 1986–November 1991
SCL 1	January 2000–December 2004
SCL 2	January 2005–December 2009
SCL 3	January 2010–December 2014
SCL 4	January 2015–December 2019

*The first time bin establishes baseline harmonic surprise. In the McGill corpus, this bin includes songs released over 16.5 years, and each remaining time bin includes songs released within 5.5 years. In the SCL corpus, each bin includes songs released with 5 years*.

For the McGill corpus, we chose to group the earliest released songs of 1958–1975 together as a baseline to compare the effects of the remaining bins. In preliminary analyses when comparing average absolute surprise measures of each Billboard quartile, we did not observe any differences across time in these measures in *Q*_1_ relative to *Q*_4_ through this period. This allowed for the resulting first time bin to serve as a substantial baseline chord distribution from which to compute a uniform measure of surprise for the remaining time bins. We attribute this lack of change in the effect of harmonic surprise on preference during this initial time-period to the establishment of a new genre: rock-n-roll.

The process resulted in four time bins for the McGill corpus (see Table 1), with 1958–1975 representing the first time bin. We then separated the SCL corpus into four time bins, each spanning 5 years of release dates, as well. We finally compared per-song average surprise and variation in surprise across sections of songs from *Q*_1_ to those from *Q*_4_.

Next, we investigated trends over time within each of the two corpora to see if absolute surprise effects on preference, contrastive surprise effects on preference, or both, were changing over time.

All of the chords of songs from the McGill Billboard corpus were transcribed by hand (Burgoyne et al., 2011). The resulting labels describe each chord “up to seventh” level. In other words, each chord label specified all notes including and up to the seventh of the chord, if appropriate. The chords for the songs of the SCL corpus were estimated by using a neural network trained to predict the root note of the chord and whether the chord is major or minor (Korzeniowski and Widmer, 2016). The difference in these two approaches to transcription results in the possibility of a slight distinction between the results in the two corpora: surprise as measured in songs of the SCL corpus is more likely to reflect pure tonality, whereas the “color” from elements other than the root and third could influence surprise in songs of the McGill corpus.

For each corpus, the relationship between harmonic surprise and preference was calculated using a method based on the approach outlined in Miles et al. (2017) and Miles (2020). Chord labels from each corpus were normalized to the key of each song. For the songs of the McGill corpus, this means that the transcribed key was identified, and then each transcribed chord was labeled according to its relationship to that key. For the SCL corpus, this means that the probable key was detected using Korzeniowski's and Widmer's Convolutional Neural Network key-detection algorithm included in the Madmom software package (Böck et al., 2016; Korzeniowski and Widmer, 2018), and then each identified chord was then labeled according to its relationship to that key.

Next, zeroth-order harmonic surprise was calculated for each chord, based on the prevalence of chords in the years from 1958 to 1975 in the case of the McGill corpus, or 2000–2004 in the case of the SCL corpus. As in Miles et al. (2017), the analysis was limited to zeroth-order harmonic surprise, which does not take into account the ordering of chords. This limitation is due to the increased statistical power necessary to determine any higher-order surprise effects in such a small corpus. Surprise was calculated by first finding *N*, the total number of unique chords in the corpus, and then for each unique chord *C*_*j*_ finding *M*_*j*_, the number of times that chord appears. This gave a total number of chords in the corpus (including repetitions) of $\Sigma_{i=1}^{N}M_{i}$. We then calculated the probability of unique chord *C*_*j*_ as in Equation (1).

(1) $$\begin{array}{l} P\left(C_{j}\right)=\frac{M_{j}}{\Sigma_{i=1}^{N}M_{i}} \end{array}$$

Given the probability of a given chord, that chord's surprise was calculated using the standard information theory equation, as shown in Equation (2).

(2) $$\begin{array}{l} S\left(C_{j}\right)=-log_{2}\left(P\left(C_{j}\right)\right) \end{array}$$

The total number of unique chords in the McGill corpus in songs from 1958 to 1975 was 348. This included chords with all twelve possible roots, and various modes and extensions for each of those root notes. The range of unique chords in songs from 2000 to 2004 in the SCL corpus, since it was based only on root and third notes, was 24. This included all twelve possible roots, each with either major or minor thirds.

Once the surprise for each chord in a piece of music was obtained, then that piece's overall absolute and contrastive surprise were found. Absolute surprise was estimated by taking the mean surprise of each chord in a song, and contrastive surprise was estimated by finding the standard deviation (SD) of mean surprise for each section of a song, with sections being calculated algorithmically according to Nieto's and Bello's Music Structure Analysis Framework (Nieto and Bello, 2016).

A uniform chord distribution of “all songs” was used in the previous analyses of Miles et al. (2017). In this analysis, we calculated surprise based on the uniform chord distribution statistics either of “1958–1975 (combined),” in the case of the McGill corpus, or of “2000–2004 (combined),” in the case of the SCL corpus.

To determine the classification of songs as “top quartile” or “bottom quartile”— *Q*_1_ or *Q*_4_—the process was slightly different for each of the two corpora. For the *McGill Billboard Corpus*, the 545 total songs were ordered by peak Billboard chart position, with number of weeks on the chart breaking any ties in chart position. The resulting 136 top-ranking songs were then classified as *Q*_1_ songs, and the 136 bottom-ranking songs were classified as *Q*_4_ songs. For the *SCL Corpus*, the songs were first broken into groups by year of release, and then further broken into groups within each year of release into genres. This was done to reduce any variability in preference for any particular genre within the charts. Genres were taken from metadata tagged by Apple Music. The resulting “year*genre” groups were then ordered by peak Billboard chart position and number of weeks on the chart. The top 25% of songs in each group were then classified as *Q*_1_ and the bottom 25% of songs in each group were classified as *Q*_4_.

## 4. Results

### 4.1. The Effect of Absolute Surprise Over Time—*Q*_1_ and *Q*_4_ Per-Song Harmonic Surprise Over Time

**Figure 1:**
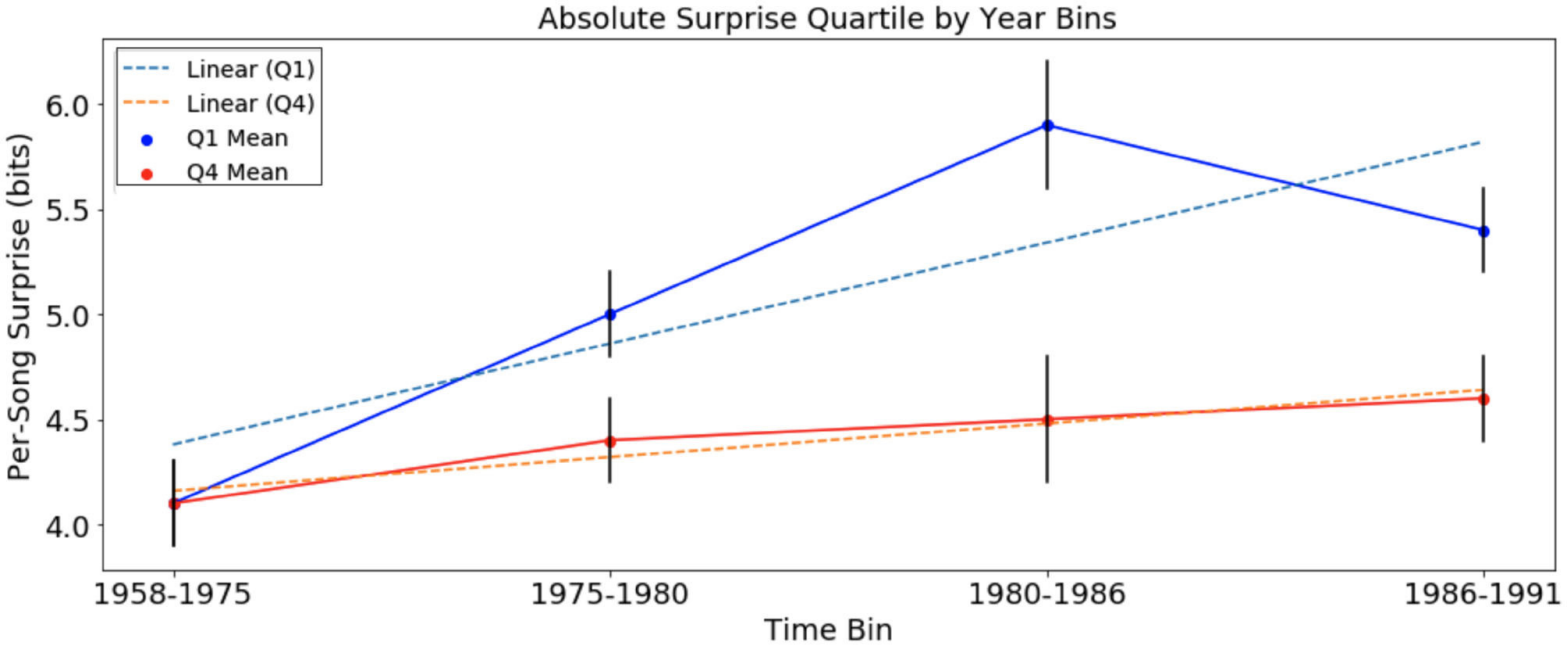
Per-song average surprise (in bits), relative to chord distribution of August 1958 to January 1975, of *Q*_1_ and *Q*_4_ separated into time bins. Error bars represent standard error. Per-song surprise rises faster in *Q*_1_ than in *Q*_4_.

**Figure 2:**
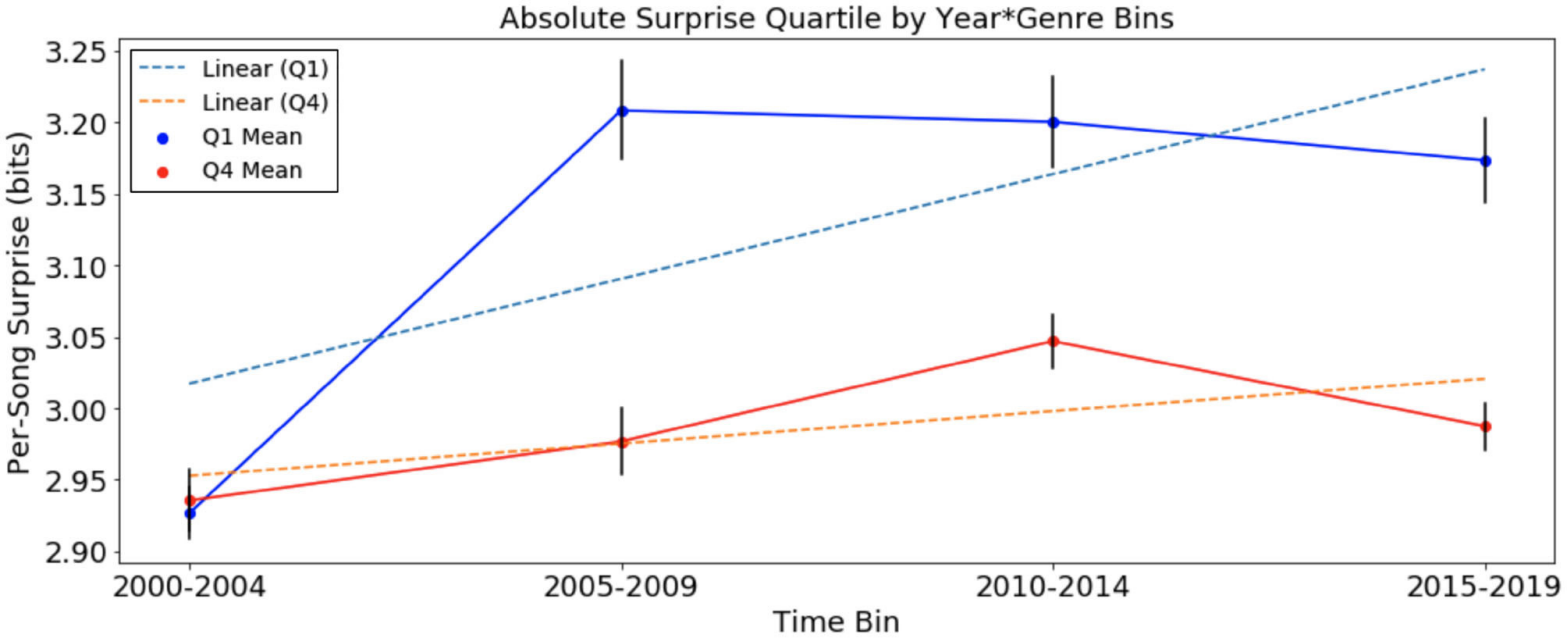
Per-song average surprise (in bits), relative to chord distribution of January 2000 to December 2004, of *Q*_1_ and *Q*_4_ (a) separated into time bins. Error bars represent standard error. Per-song surprise rises faster in *Q*_1_ than in *Q*_4_.

We looked at per-song average surprise in the four newly defined time bins of each corpus, across top and bottom quartiles. We then computed linear regression lines best fitting the *Q*_1_ and *Q*_4_ data over time. The results of the analyses are presented in Figures 1, 2 below.

In the analysis of absolute surprise over time in the McGill corpus, with per-song average surprise calculated using the chord distribution of bins 1958–1975 (and excluding newly introduced chords), there were significant upward trends in *Q*_1_ (Jonckheere-Terpstra, *p* < 0.001) and in *Q*_4_ (Jonckheere-Terpstra, *p* < 0.05). This suggests an increase in surprise over time for songs of both quartiles. Tests showed that while there was no significant difference in per-song average surprise between *Q*_1_ and *Q*_4_ in time-bin “1958–1975,” per-song average surprise was significantly higher among the three remaining time bins (*t*-test, *Q*_1_ mean = 5.46; *Q*_4_ mean = 4.78, *p* < 0.01). In the data of Figure 1, we observed that the average rate of change for songs in *Q*_1_, +0.096 bits/year, was three times the trend in *Q*_4_, +0.032 bits/year. In order to test the significance of the slopes calculated for trends over time in *Q*_1_ and *Q*_4_, we also tested the null hypothesis that slopes are equivalent using the regression slopes test provided by Zaiontz (2013). Equation (3) below gives the statistic *t* that was calculated using the slopes obtained from linear regression of surprise data for *Q*_1_ and *Q*_4_. The test was normalized by using the standard error of the slope for *Q*_1_ and *Q*_4_, *s*_*Q*_1__, and *s*_*Q*_4__, respectively. In this equation, *n*_1_ and *n*_2_ represent the number of songs in *Q*_1_ and *Q*_4_, respectively, and *T* represents the student's t distribution.

(3) $$\begin{array}{l} t=\frac{slope\left(Q_{1}\right)-slope\left(Q_{4}\right)}{\sqrt{s_{Q_{1}}^{2}+s_{Q_{4}}^{2}}}\sim T\left(n_{1}+n_{2}-4\right) \end{array}$$

For the data in Figure 1, we obtain a p-value of 1.8 × 10^−12^. This suggests that the increase in absolute surprise for *Q*_1_ songs was more pronounced than that of *Q*_4_ songs in the McGill corpus.

In the analysis of absolute surprise over time of the SCL corpus, with per-song average surprise calculated using the chord distribution of 2000–2004, there were upward trends in *Q*_1_ and *Q*_4_. The average rate of change for songs in *Q*_1_, +0.0146 bits/year, is also greater than three times the rate in *Q*_4_, +0.0046 bits/year. This suggests an increase in surprise over time for songs of both quartiles. With the same statistical test comparing the slopes of the two trend lines for the SCL corpus data, we obtained a p-value of 3 × 10^−11^, suggesting that the increase in *Q*_1_ songs was more pronounced than that of *Q*_4_ songs in the SCL corpus. Note that the scale of calculated surprise in songs of the SCL corpus is distinct from that of the McGill corpus. This is the result of the different methodology in calculating surprise: in the McGill corpus, chords were transcribed by humans up to the seventh tone, while in the SCL corpus, chords were algorithmically determined only at the root and third tones. This resulted in far fewer unique chords in the SCL corpus than in the McGill corpus (24 and 348, respectively), thereby lowering the overall surprise values for each chord in the SCL corpus.

### 4.2. Effect of Contrastive Surprise Over Time: Variation in Surprise Among Sections Within Songs Over Time

**Figure 3:**
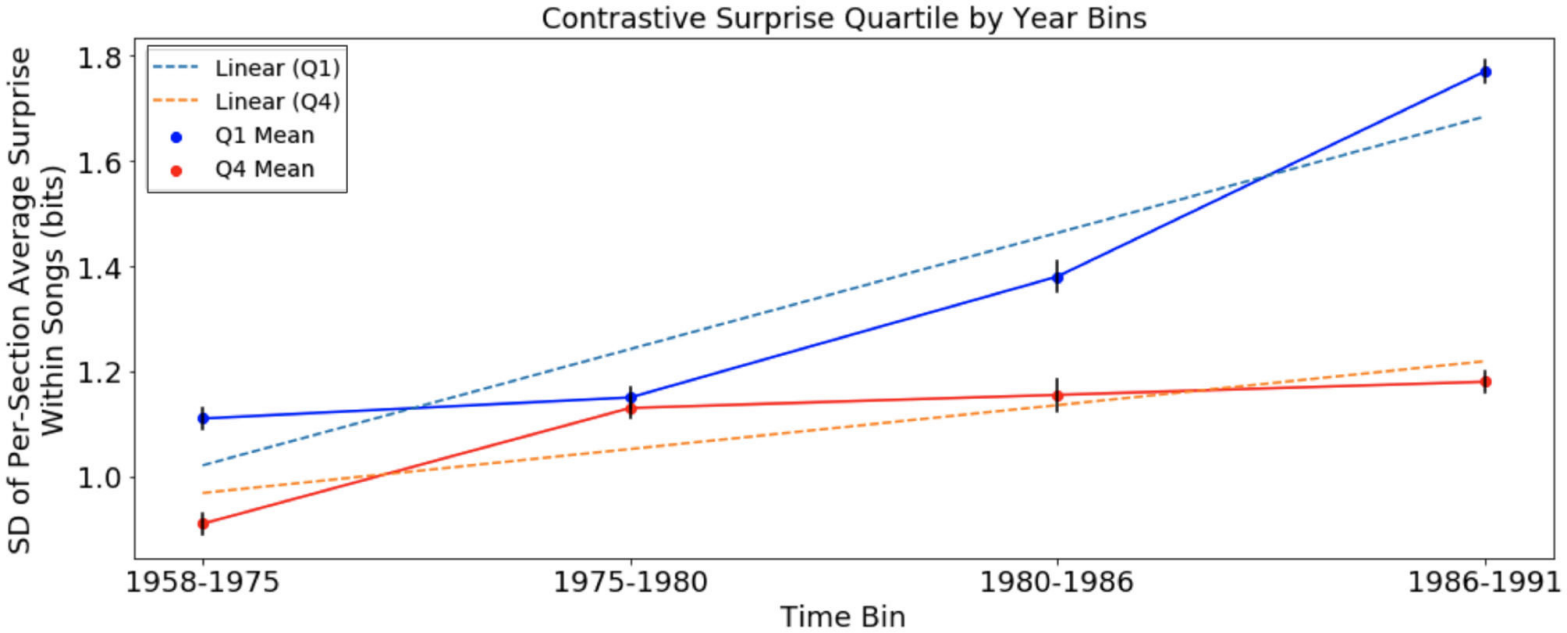
Variation of harmonic surprise across sections of songs, with harmonic surprise calculated using chord distribution of August 1958 to January 1975, of *Q*_1_ and *Q*_4_, separated into time bins. Error bars represent standard error. Variation of surprise across sections rises faster in *Q*_1_ than in *Q*_4_.

**Figure 4:**
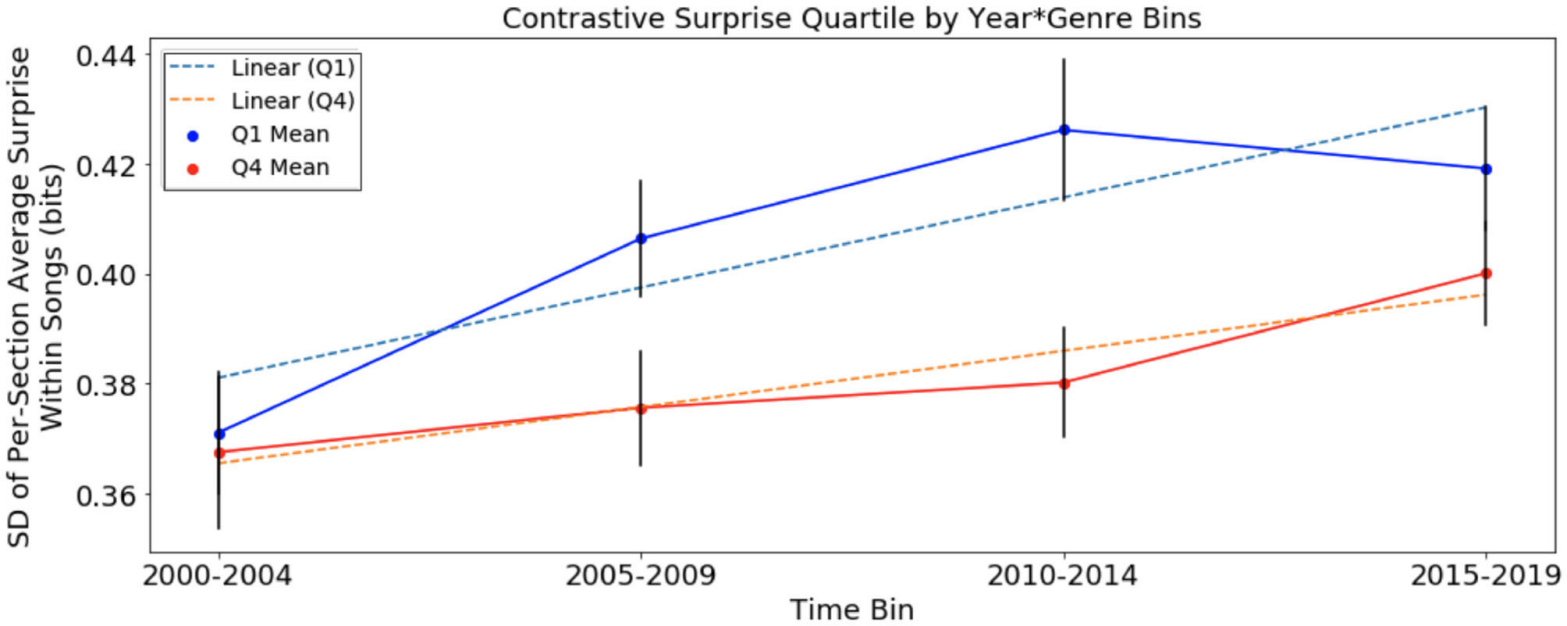
Variation of harmonic surprise across sections of songs, with harmonic surprise calculated using chord distribution of January 2000 to December 2004, of *Q*_1_ and *Q*_4_, separated into time bins. Error bars represent standard error. Variation of surprise across sections rises faster in *Q*_1_ than in *Q*_4_.

Next, we looked at variation of surprise across sections within *Q*_1_ and *Q*_4_ songs in the same time bins used in the previous section with surprise computed using the chord distribution of the original 1958–1975 distribution and 2000–2004 distribution for McGill and SCL corpora, respectively. The results are presented below in Figures 3, 4.

In this analysis, there was an increase in variation across sections of songs in both *Q*_1_ and *Q*_4_ (Jonckheere-Terpstra, *Q*_1_: *p* < 0.001; *Q*_4_: *p* < 0.05). The rate of change for songs in *Q*_1_ at +0.044 bits/year is more than twice the slope of the trend in *Q*_4_ at +0.0166 bits/year. Tests showed that while there was no significant difference in variation across sections between *Q*_1_ and *Q*_4_ in time bin 1958–1975, variation was significantly higher among time bins 2–4 (*t*-test, *Q*_1_ mean = 1.45; *Q*_4_ mean = 1.21, *p* < 0.05). Additionally, *p* < 1 × 10^−100^ for the slope test suggests that the increase in *Q*_1_ songs was more pronounced than that of *Q*_4_ songs in the McGill corpus.

In the analysis of contrastive surprise over time in Figure 4, with variation of surprise across sections using the chord distribution of 2000–2004 in the SCL corpus, there were upward trends in *Q*_1_ and *Q*_4_. The rate of change in *Q*_1_ of +0.0032 bits/year is approximately 1.5 times the rate of change in *Q*_4_ of +0.002 bits/year. This suggests an increase in surprise over time for songs of both quartiles. As we did in the previous analysis, we test the null hypothesis that slopes are equivalent and obtain a *p*-value of 0.008. This suggests that the increase in contrastive surprise for *Q*_1_ songs was significantly more pronounced than that of *Q*_4_ songs in the SCL corpus. Note that the scale of these results from songs of the SCL corpus is again smaller, due to the much smaller number of possible unique chords.

### 4.3. New Chords Introduced in *Q*_1_ and *Q*_4_ Songs

**Figure 5:**
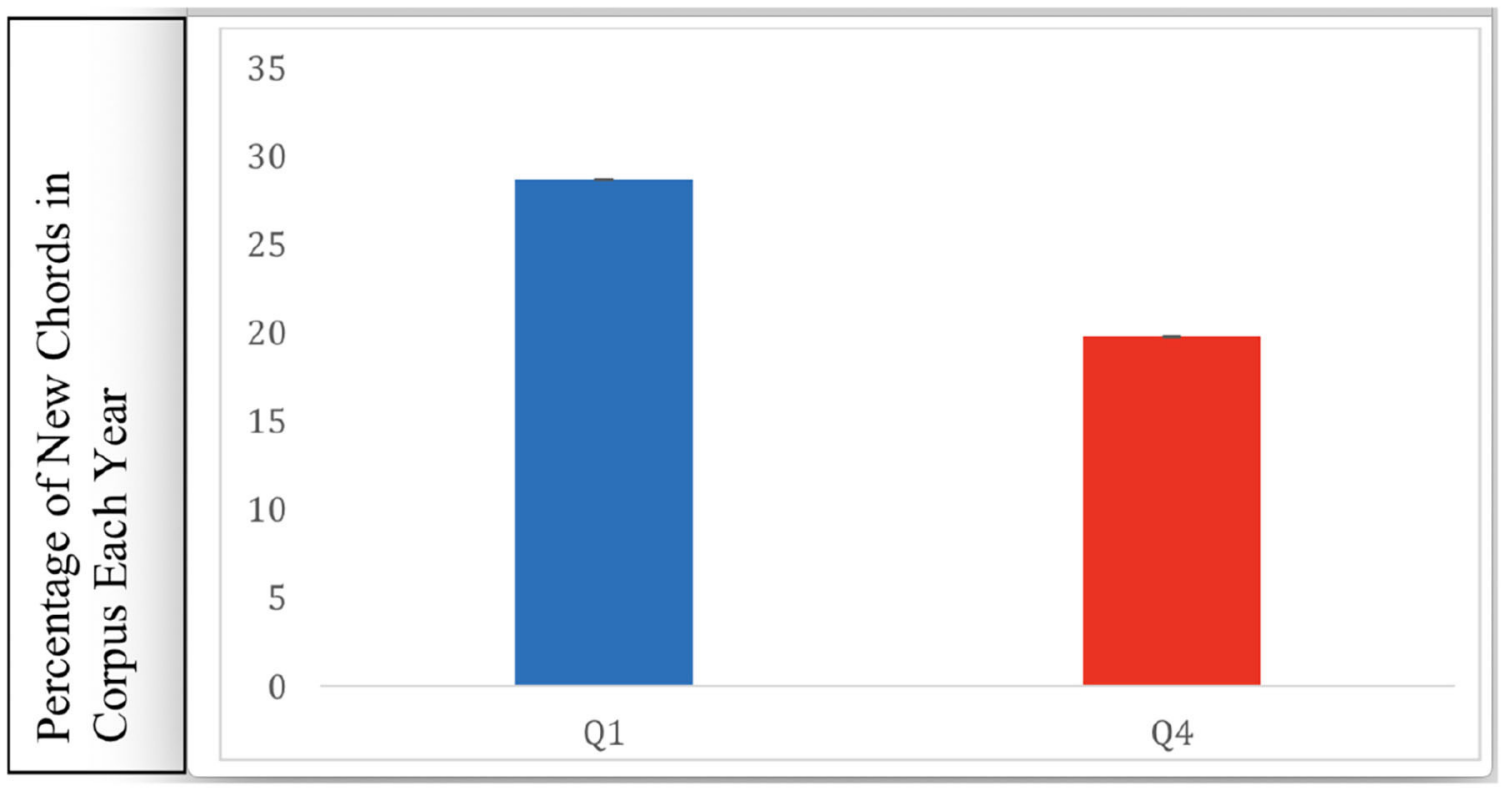
Percentage of chords that appear over the years from 1959 to 1991, that had not appeared in songs of previous years, in *Q*_1_ and *Q*_4_. Error bars represent standard error. There were significantly more novel chords featured in *Q*_1_ songs.

In the McGill Billboard corpus, “up to seventh” labels of chords included myriad permutations of each chord based on the possible 12 roots and 12 thirds. This was not an issue with the SCL corpus, which only included root and third notes in its chord labels. By using a chord distribution, in the McGill corpus analyses, that reflects only songs of 1958–1975, we failed to account for any surprise effects due to new chords that might be introduced into the distribution over time. Chords being introduced for the first time in the corpus are likely to be highly harmonically unexpected and could significantly contribute to any effects of harmonic surprise on preference. To examine the relative contribution to surprise of new chords, we examined the prevalence of chords in each quartile that appeared in years 1959 to 1991 that had not previously appeared in any year (Figure 5). This analysis showed that 28.7% of newly introduced chords appeared in *Q*_1_ songs, and 19.8% of newly introduced chords appeared in *Q*_4_ songs. New chords were found to appear significantly more frequently in *Q*_1_ than in *Q*_4_ (χ^2^ = 133.5, df = 1, *p* < 0.001).

## 5. Discussion

### 5.1. Findings and Their Support for the Proposed Hypotheses

In the statistical corpus analyses reported in Miles et al. (2017), we found evidence that the brain uses both absolute- and contrastive-surprise strategies in determining preference for popular songs. In the present study, we tested whether absolute and contrastive harmonic surprise effects on preference varied over time across the 33 years of the McGill corpus and across the 20 years of the SCL corpus.

Tests of trend showed that in both corpora, *Q*_1_ and *Q*_4_ average per-song surprise increased across consecutive time bins when surprise was calculated using the chord distribution of the first time bin, and that *Q*_1_ average per-song surprise increased at a significantly greater rate. Also in both corpora, tests of trend showed that variation in surprise across sections of *Q*_1_ and *Q*_4_ songs increased over time, and that variation in surprise across sections of *Q*_1_ songs increased at a significantly greater rate. These results rule out the null hypothesis that absolute and contrastive harmonic surprise effects on music preference do not vary over time.

Rather, these results support the Inflationary-Surprise Hypothesis: it appears that the effects of harmonic surprise on music preference have to be more pronounced over time to get the same effect. The force driving harmonic surprise upward in the Hot 100 chart, and more forcefully upward in *Q*_1_ songs specifically, could be due to the statistical learning of harmonic regularities. Expectations for these regularities could be learned early in life, during a critical window for developing models of tonality. The window of consumers driving performance of songs on the chart features a cycle of successive cohorts of 13- to 19-year old listeners. These listeners are likely to develop their baseline expectations from the preferred music released in the past, and because of the relationship between moderate increases in harmonic surprise and preference, the cycle results in preferred music that increases in harmonic surprise relative to a fixed composition of chords.

Successful musicians from the present use chords that are surprising not just for the current moment but also relative to the past. These successful musicians also keep introducing new chords. Consequently, the probability distribution of chords is ever changing, such that musicians must create new surprise to accomplish the same level of preference, leading to an inflationary effect.

Another interesting result is that in the McGill corpus, per-song average surprise (Figure 1), the rise of *Q*_1_ surprise seems to level off around six bits. There is a corresponding plateau effect of per-song average surprise in the SCL corpus around 3.2 bits. This could be evidence of a ceiling effect for absolute surprise. The data here are not sufficient to test this hypothesis. It is possible that this is related to an “inverted-U” effect of the relationship between complexity and pleasure (Berlyne, 1973). It is also possible that the inflationary effect of the relationship between harmonic surprise and preference can only go so far, and the trend is not infinitely sustainable. Future research might be useful in exploring whether there is an ideal range of harmonic surprise in popular music, such that too much or too little harmonic surprise is inversely related to pleasure.

When calculated relative to the chord distribution statistics from songs released from August 1958 to February 1975 in the McGill corpus, and when calculated relative to the chord distribution statistics from songs released from 2000 to 2004 in the SCL corpus: per-song average surprise in *Q*_1_ and variation in surprise across sections of *Q*_1_ songs were shown to increase more over time than the corresponding measures of *Q*_4_ songs. Additionally, chords introduced for the first time in a given year were more likely to appear in a *Q*_1_ song than in a *Q*_4_ song in the McGill corpus. Taken together, these findings are consistent with the Surprise-Inflation Hypothesis.

Evidence for measurable increases of harmonic surprise over time and for apparent increases over time in the magnitude of measured surprise advantages in preferred music is probably linked to how schematic information about musical systems is acquired. Capacity for perceiving fundamental pitch features in music, such as octave equivalence, are thought to be evolutionarily conserved, extending to other species (Greenwood, 1997). Higher-level aspects of music processing such as tonality, however, have been shown to be statistically learned through exposure (Tillmann et al., 2000; Loui and Wessel, 2008). In addition, structural expectations have been demonstrated to be learned through regularities within auditory sequences (Saffran et al., 1999; Tillmann and Poulin-Charronnat, 2010). While there is empirical evidence of statistical learning (even within the time frame of a behavioral experiment) of schematic regularities within artificial music systems, there is less evidence of shifts in higher-level expectations based on tonality in ecologically valid music. A notable exception, however, is presented in Rohrmeier and Widdess (2012), where exposure to a novel tonal system of regularities impacted subsequent expectations by participants. Investigations into tonality in Western music (e.g., Krumhansl and Keil, 1982; Tillmann et al., 2000), approach its system of tonality as a relatively fixed hierarchy. The finding of these differing surprise measures over time, however, is evidence of non-static harmonic expectations within Western popular music, as well as shifts in preference for various harmonic elements within it. Such shifts in preference are consistent with several components of the framework of how aesthetic values are learned over time presented in Aleem et al. (2020). These components include the shaping of reward value according to probabilistic information from exposure to stimuli and a “peak-shift” effect marked by the exaggeration of desirable features.

## 6. Conclusion

In an examination of the relationship between harmonic surprise and preference in popular music over the years, we found that surprise relative to a fixed distribution of chords seems to increase over time, and that this increase is significantly more pronounced in preferred songs. Such dynamic harmonic expectations highlight the interactions between individual listeners and musicians with the culture around them. The Surprise-Inflation Hypothesis raised by the results presented here suggests that the brain's craving for surprise causes continuous changes in harmonic distributions in popular music. A musician exposed to changes advanced by other musicians must innovate to be successful. It could be that musicians, learning from the success of high-surprise songs from one year, end up producing more high-surprise songs the next year. This could be an explicit strategy to improve on the part of musicians, rather than an implicit change in expectation on the part of the listeners. However, listeners' preferences change as a result, forcing musicians to incorporate further changes. Hence, the inherent craving for surprise in each of us may push our entire culture in an endless evolution of musical preferences.

## Data Availability Statement

The raw data supporting the conclusions of this article will be made available by the authors, without undue reservation.

## Author Contributions

SM was involved in the design of the project, developing, and using programming scripts for data analysis, interpreting results, writing, and revising the manuscript. SB was involved in the data analysis, implementation of neural networks, and writing the manuscript. DG was involved in data collection, interpreting the results, and writing the manuscript. DR was involved in the design of the project, interpreting results, writing, and revising the manuscript. NG was involved in the design of the project, devising the formulas, interpreting results, writing, and revising the manuscript. All authors contributed to the article and approved the submitted version.

## Conflict of Interest

The authors declare that this study received funding from Secret Chord Laboratories. The authors are employees of SCL and proprietary algorithms belonging to SCL were used to analyze the data.
